# Molecular engineering approaches to half-life extension of therapeutic biomolecules

**DOI:** 10.3389/fphar.2026.1778569

**Published:** 2026-04-10

**Authors:** Yuli Wang, Junliang Chang, Jinghui Liu

**Affiliations:** Changchun Institute of Biological Products Co., Ltd, Changchun, China

**Keywords:** fusion proteins, long-acting therapeutic biomolecules, molecular engineering, pharmacokinetics, unstructured polypeptides

## Abstract

Therapeutic biomolecules are widely used to treat various diseases, but their clinical efficacy is often limited by short *in vivo* half-lives due to rapid renal filtration, proteolytic degradation, and immune clearance. Short half-life leads to frequent dosing, fluctuation in blood drug concentration, and increased treatment costs, severely limiting clinical efficacy. To address these challenges, various half-life extension strategies have been developed, including chemical conjugation (e.g., PEGylation), physical delivery systems (e.g., microspheres), and genetic/fusion approaches. This review provides a comprehensive narrative analysis of molecular engineering methods, discussing the rational design to directly optimize the drug molecule itself or fuse it with long-acting carriers to significantly extend its circulation time. A systematic comparison of several approaches is presented to guide rational strategy selection. By synthesizing current knowledge and recent advances, this review serves as a practical resource for researchers and drug developers navigating half-life extension technologies.

## 1 Introduction

Recombinant therapeutic biomolecules represent a class of biopharmaceuticals engineered via genetic modification to replicate or augment the biological activity of endogenous human proteins. These therapeutic agents are widely used to treat various diseases, including diabetes (e.g., insulin) (Pampanelli et al., 1995; Nauck et al., 2021), anemia (e.g., erythropoietin) (Eschbach and Adamson, 1989; Bunn, 2013; Heo, 2024), autoimmune disorders (e.g., monoclonal antibodies) (Cvetkovic and Keating, 2002; Dhillon, 2021; Cook and Schafer, 2020; Frampton and Lyseng-Williamson, 2009), and cancers (e.g., cytokines) (Balkwill, 2009; Brinkmann et al., 2001; Donà et al., 2021; Kontermann, 2005; Eltarhoni et al., 2022). In contrast to small-molecule drugs, recombinant proteins are large, complex molecules with high specificity and potency. Nevertheless, their clinical application is often constrained by inherent challenges related to stability, immunogenicity, and suboptimal pharmacokinetic profiles.

A major limitation in the clinical utility of many therapeutic biomolecules is their short *in vivo* half-life. This rapid clearance is primarily attributed to their relatively low molecular mass, which often falls below the renal glomerular filtration threshold, rendering them susceptible to efficient renal elimination. Additionally, these molecules are prone to proteolytic degradation, hepatic uptake, and potential recognition by the immune system, further accelerating their systemic clearance. For instance, native glucagon-like peptide-1 (GLP-1) exhibits an exceptionally short half-life of approximately 2 min (Nauck, 1998), while interferon-β1b lasts merely 4–15 h after intravenous injection (Harari et al., 2015).

The suboptimal pharmacokinetic profile of therapeutic biomolecules results in several clinically significant limitations. Frequent dosing requirements—often necessitating daily or even multiple daily injections—compromise patient convenience and adherence. Moreover, rapid systemic clearance results in pronounced fluctuations in drug concentrations, characterized by peak-and-trough patterns that may lead to transient subtherapeutic exposure or concentration-dependent toxicity. These pharmacokinetic shortcomings also contribute to increased treatment burden and elevated healthcare costs, as higher or more frequent doses are necessary to maintain efficacy. In chronic conditions such as diabetes, hemophilia, and growth hormone deficiency, the short half-life of therapeutic proteins undermines sustained disease control and compromises long-term clinical outcomes.

To overcome these challenges, a range of half-life extension strategies have been developed, including PEGylation, Fc fusion, albumin binding, and fusion with unstructured polypeptides (e.g., XTEN, PAS). These approaches are designed to enhance proteolytic stability, reduce renal clearance, and prolong systemic exposure, ultimately enabling less frequent dosing and improving patient quality of life.

While numerous comprehensive reviews have previously addressed half-life extension strategies for protein therapeutics and have provided valuable insights into their respective fields, there remains a paucity of systematic comparisons that position genetic fusion-based approaches as a central focus while concurrently evaluating chemical and physical strategies.

**FIGURE 1:**
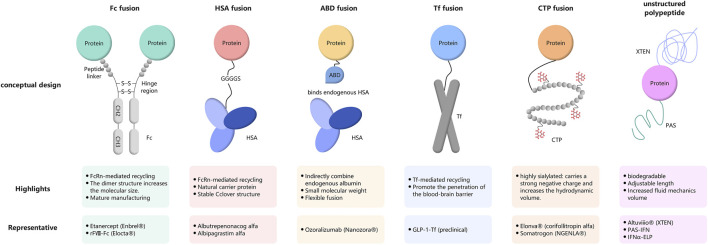
Schematic representation of genetic/fusion platforms for half-life extension. For each platform, the molecular architecture is depicted (top), key mechanistic features and advantages are highlighted (medium), and representative therapeutic products are listed (bottom). Fc fusion exploits FcRn-mediated recycling and dimeric structure; HSA fusion leverages endogenous albumin recycling; ABD fusion indirectly binds circulating albumin; Tf fusion utilizes transferrin receptor-mediated recycling; CTP fusion increases hydrodynamic volume through sialylation; unstructured polypeptides (XTEN, PAS) provide biodegradable, size-tunable half-life extension.

This review provides an updated and integrated perspective with the following distinctive contributions: (i) it offers a comprehensive examination of genetic/fusion strategies—including Fc fusion, HSA fusion, non-structured polypeptides (XTEN, PAS), and site-directed mutagenesis—as the primary half-life extension platform (Figure 1); (ii) by consolidating these mechanistically related yet distinct approaches, it illuminates the design rationale, versatility, and clinical potential of genetic fusion platforms; and (iii) it systematically compares these genetic approaches with chemical conjugation (e.g., PEGylation, fatty acid acylation) and physical delivery systems (e.g., microspheres, liposomes) within a unified framework, highlighting their respective mechanisms, advantages, and limitations. By integrating these dimensions, this review aims to serve as a comprehensive resource for researchers and drug developers navigating the increasingly diverse landscape of half-life extension technologies, with a particular focus on the versatility and drug development success of genetic/fusion platforms.

## 2 Mechanisms for extending the half-life of therapeutic biomolecules

**FIGURE 2:**
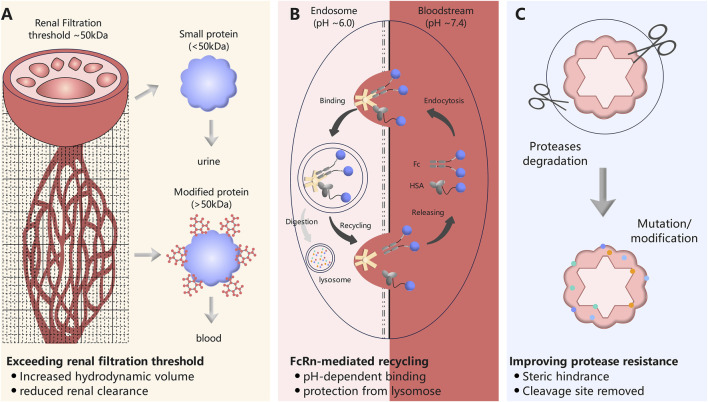
Schematic representation of three complementary mechanisms for extending the half-life of protein therapeutics. **(A)** Increasing hydrodynamic volume to exceed the renal filtration threshold. Unmodified small proteins (<50 kDa) are readily filtered through the glomerulus into urine, whereas conjugation to bulky carriers (e.g., PEG, Fc, unstructured polypeptides) increases molecular size, preventing renal clearance. **(B)** Harnessing FcRn-mediated recycling to salvage proteins from lysosomal degradation. Following cellular uptake, Fc-fusion proteins bind to FcRn in the acidic endosomal compartment (pH 6.0) and are recycled back to the cell surface, where neutral pH (7.4) triggers release. Unbound proteins proceed to lysosomes for degradation. **(C)** Enhancing intrinsic stability against proteolytic cleavage. Site-directed mutagenesis removes protease recognition motifs, and conjugation to polymers (PEG, glycosylation) creates steric hindrance that shields the protein from protease access.

The pharmacokinetic profiles of therapeutic biomolecules are governed by a complex interplay of physiological processes, including renal clearance, proteolytic degradation, and receptor-mediated elimination. Strategies to extend systemic half-life are therefore rationally designed to modulate one or more of these key determinants. As illustrated in Figure 2, the principal mechanisms of action can be categorized into three complementary approaches: (i) increasing the hydrodynamic volume to reduce renal filtration; (ii) engaging the neonatal Fc receptor (FcRn) recycling pathway to salvage proteins from lysosomal degradation; and (iii) enhancing intrinsic stability against proteolytic cleavage. This section provides a mechanistic overview of these fundamental principles, which form the basis for the diverse half-life extension technologies discussed subsequently.

### 2.1 Exceeding the renal filtration threshold

The kidneys constitute a primary elimination pathway for protein therapeutics, with the glomerular capillary wall functioning as a size-selective filtration barrier. This barrier efficiently restricts the passage of macromolecules based on their hydrodynamic radius, while permitting the filtration of smaller proteins and peptides into the urinary space. The molecular weight threshold for renal filtration has been empirically established to lie within the range of approximately 36–44 kDa (Sigurjonsson et al., 2024). Consequently, recombinant therapeutic biomolecules with molecular dimensions below this critical threshold are subject to rapid glomerular filtration and subsequent elimination from systemic circulation, resulting in abbreviated half-lives and suboptimal pharmacokinetic profiles. To circumvent this pathway, the main strategy is to increase the apparent hydrodynamic radius of the therapeutic biomolecule well above this threshold. This can be achieved by combining it with inert, bulky carrier molecules without altering the therapeutic effect. Examples include the covalent attachment of PEG chains or the genetic fusion to a large, soluble protein domain.

### 2.2 Harnessing FcRn-mediated recycling

The exceptionally prolonged systemic half-life of endogenous immunoglobulin G (IgG) antibodies, which persists for approximately 21 days in humans, cannot be attributed solely to their molecular size exceeding the renal filtration threshold. Rather, this pharmacokinetic advantage arises from an active recycling mechanism mediated by the neonatal Fc receptor (FcRn) (Vidarsson et al., 2014). This receptor serves a homeostatic function by salvaging IgG and albumin from the lysosomal degradation pathway, thereby maintaining their abundance in the circulation. Harnessing this natural recycling machinery has emerged as a pivotal strategy for extending the half-life of recombinant protein therapeutics.

The molecular basis of FcRn-mediated recycling involves pH-dependent binding dynamics. Circulating IgG is internalized into cells via fluid-phase pinocytosis and trafficked to acidic endosomal compartments. Within these endosomes, where the pH is approximately 6.0, the FcRn binds with high affinity to the Fc domain of IgG. This interaction sequesters IgG from the degradative pathway, directing it instead to recycling endosomes that transport the FcRn-IgG complex back to the cell surface. Upon exposure to the neutral pH of the bloodstream (approximately 7.4), a conformational change disrupts the binding affinity, resulting in the release of intact IgG back into systemic circulation (Roopenian and Akilesh, 2007). Proteins that fail to engage FcRn proceed through the endosomal pathway to lysosomes, where they undergo proteolytic degradation. The common strategies include Fc fusion, albumin fusion and albumin binding, etc.

### 2.3 Improving protease resistance

Beyond renal clearance and FcRn-mediated catabolism, proteolytic degradation represents a significant elimination pathway for therapeutic biomolecules. Proteases present in the blood, liver, and at sites of administration can cleave peptide bonds, inactivating the therapeutic agent. This approach focuses on the molecular structure of the therapeutic protein itself, aiming to make it a less suitable substrate for proteolytic enzymes.

Proteolytic degradation can be mitigated through several complementary strategies. Site-directed mutagenesis enables the selective abrogation of protease cleavage by targeting and modifying surface-exposed recognition motifs. Such substitutions are carefully designed to preserve the protein’s structural integrity and functional activity. To counter exopeptidase-mediated degradation, which proceeds progressively from the N- or C-terminus, terminal modifications—including peptide cyclization or the attachment of protective blocking groups—can be employed to confer robust resistance. Additionally, conjugation to macromolecular carriers such as polyethylene glycol (PEG) or other proteins offers a bifunctional mechanism of extension: it increases the hydrodynamic radius to reduce renal clearance while simultaneously erecting a steric shield that restricts protease access to the protein surface, thereby providing a synergistic enhancement of *in vivo* half-life.

## 3 Protein structure alteration

The structure of a protein is organized into four hierarchical levels, each of which exerts a distinct influence on its pharmacokinetic (PK) behavior. The primary structure, denoting the linear sequence of amino acids, dictates the protein’s susceptibility to proteolytic cleavage; specific sequence motifs serve as recognition sites for endopeptidases in the bloodstream, thereby governing the metabolic stability of the drug. The secondary structure includes local folding patterns such as α-helices and β-sheets, which contribute to the protein’s conformational rigidity and resistance to denaturation. The tertiary structure represents the complete three-dimensional folding of a single polypeptide chain, forming ligand-binding domains and active sites. The integrity of the tertiary structure is crucial for maintaining solubility and preventing aggregation, as misfolded proteins are rapidly cleared or may provoke unwanted immunogenicity, accelerating clearance. Finally, when two or more independently folded polypeptide chains associate through non-covalent interactions, they form a quaternary structure. This level is of particular pharmacological importance for half-life extension: the assembly of subunits increases the protein’s hydrodynamic radius, effectively reducing renal filtration. Modifications to amino acid composition or structure—such as reducing unstable residues, enhancing protease resistance, and improving structural stability—can effectively extend the plasma half-life of recombinant therapeutic biomolecules.

### 3.1 Amino acid substitution

Certain metabolically unstable amino acids present in protein and peptide therapeutics can significantly reduce their plasma half-life. Therefore, replacing such unstable residues represents an effective strategy for extending the drug’s circulation time.

Glargine insulin, the first clinically established long-acting insulin analog, exemplifies a strategy focused on absorption-site PK prolongation. Its prolonged action is achieved through two key modifications: a di-arginine extension at the C-terminus of the insulin B-chain and a glycine substitution in the A-chain. This engineered structure enables glargine to precipitate subcutaneously after injection, followed by gradual dissolution and release into circulation, thereby maintaining stable blood glucose levels for up to 24 h (Home, 1999). Therefore, it is achieved not by altering the drug-receptor interaction (pharmacodynamics, PD), but by creating a slow-release absorption phase, which extends the systemic exposure time without changing the intrinsic activity of the insulin molecule itself.

Moreover, GLP-1, a promising therapeutic candidate for type 2 diabetes (T2D), is limited by a short plasma half-life, primarily due to rapid degradation by dipeptidyl peptidase-IV (DPP-IV) and renal clearance. By introducing a cysteine substitution in the native GLP-1 sequence to create a disulfide bridge, a stable homodimeric analog can be generated. This engineered dimer significantly extends the biological half-life and enhances the *in vivo* stability of GLP-1 (Li et al., 2011). Notably, this dimerization strategy primarily enhances pharmacokinetic stability rather than altering the intrinsic agonistic potency (pharmacodynamics) of the GLP-1 peptide at the receptor.

In addition, the long-acting human fibroblast growth factor 21 (FGF21) fusion protein has been reported to undergo C-terminal degradation, leading to fragmented species that impair its bioactivity. The introduction of the V169L mutation at this specific degradation site represents a direct enhancement of molecular stability (PK). By shielding the protein from proteolytic cleavage, the V169L mutation increases the mean residence time of the intact, fully active molecule in circulation. Consequently, this pharmacokinetic optimization leads to a marked improvement in the desired pharmacodynamic response—protection against hepatocyte apoptosis (Ji et al., 2024a).

The examples above illustrate that site-directed mutagenesis can extend a protein’s therapeutic window through either pharmacokinetic or pharmacodynamic mechanisms, or a combination of both. Site-directed mutagenesis at key positions can effectively alter the properties of therapeutic biomolecules, whereas identifying such critical sites through random mutagenesis remains a relatively inefficient and uncertain approach. In addition, the impact of site-directed mutagenesis on the immunogenicity of protein therapeutics is highly context-dependent and cannot be generalized. For instance, mutations that alter the isoelectric point to achieve prolonged action, as exemplified by glargine insulin, exhibit immunogenicity profiles comparable to those of native human insulin. However, if mutations induce protein aggregation or directly perturb the three-dimensional conformation, they may significantly increase the risk of immunogenicity.

### 3.2 Protein truncation

The strategy of truncating proteins to extend their therapeutic half-life, while seemingly paradoxical, represents a deliberate and effective molecular engineering approach. The underlying rationale involves the removal of structural domains responsible for mediating rapid clearance, receptor-mediated uptake, or intrinsic instability, thereby retaining the core functional module while improving the overall pharmacokinetic profile.

The most established example of this strategy is the engineering of reteplase from alteplase (tissue plasminogen activator). This modification entails the deletion of specific N-terminal domains—namely the Finger, Epidermal Growth Factor (EGF), and Kringle 1 domains—which are known to mediate rapid hepatic clearance. Their removal substantially reduces the rate of liver uptake, directly lowering systemic clearance and extending the plasma half-life of the thrombolytic agent (Wooster and Luzier, 1999).

### 3.3 Cyclization

Backbone cyclic peptides and proteins have garnered significant interest for their enhanced resistance to proteolytic degradation. Utilizing cyclization—including intramolecular ring formation or the creation of stable cyclic structures—to extend the *in vivo* half-life of therapeutic proteins represents a critical strategy in therapeutic biomolecule engineering. This design primarily achieves prolonged activity by reducing the accessibility of proteolytic cleavage sites, enhancing structural rigidity, and decreasing renal clearance. Sortase-mediated protein cyclization offers a strategy to enhance conformational stability without introducing exogenous modifications, thereby mitigating the potential immunogenicity risks associated with extrinsic chemical conjugation. For example, the covalent cyclization of single-chain Fv antibodies (scFvs), achieved by sortase A-mediated linkage of their N- and C-termini, effectively constrains the dynamic movement of the VH and VL domains. This structural restraint significantly suppresses improper intermolecular interactions, thereby overcoming the inherent aggregation propensity of scFvs while maintaining their antigen-binding activity, enabling scFvs becoming the next-generation therapeutics (Yamauchi et al., 2019). Beyond sortase-mediated ligation, site-specific protein cyclization can also be accomplished through alternative approaches, including intein-mediated cyclization and click chemistry involving non-canonical amino acids.

## 4 Fusion proteins

Long-acting strategies based on fusion protein expression involve genetically fusing a therapeutic protein or peptide to an endogenous carrier protein with an intrinsically long plasma half-life. This biosynthetic approach allows host cells to directly produce a single, stable fusion protein with significantly extended pharmacokinetic properties. The most commonly employed carrier proteins include the Fc fragment of immunoglobulin G, human serum albumin (HSA), and transferrin, each of which exploits distinct recycling mechanisms to prolong systemic exposure. CTP (Carboxyl terminal peptide) fusion is another widely used strategy for extending the half-life of therapeutic proteins. This approach involves genetically fusing the therapeutic biomolecule to a CTP sequence that is typically derived from the beta subunit of human chorionic gonadotropin (hCG). The CTP moiety enhances sialylation of the fusion protein and increases its hydrodynamic radius, thereby prolonging serum half-life through reduced renal clearance and potential engagement with salvage receptors.

### 4.1 Fc

The neonatal Fc receptor for IgG (FcRn) contributes to effective humoral immunity by recycling IgG and extending its half-life in the circulation (Roopenian and Akilesh, 2007). Similarly, fusion of recombinant therapeutic biomolecules to the IgG Fc fragment exploits this FcRn-mediated salvage pathway while also increasing molecular weight to reduce renal filtration, thereby extending therapeutic exposure. In 1989, Capon et al. constructed the first therapeutic Fc fusion protein by fusing the extracellular domain of the CD4 molecule to the Fc region of human IgG1. This protein specifically bound to the envelope glycoprotein of the human immunodeficiency virus (HIV) and inhibited HIV infection of cells (Capon et al., 1989). Over the next few decades, an increasing number of Fc fusion proteins were developed.

Different IgG subtypes exhibit varying affinities for FcRn, with IgG1 being the most commonly employed subtype in current recombinant Fc fusion protein therapeutics. To further enhance pharmacokinetics, genetic engineering is utilized to introduce site-specific mutations into the Fc region, thereby increasing its affinity for FcRn and generating longer-lasting variants. A prominent example is the YTE mutation (M252Y, S254T, T256E) in motavizumab (an anti-RSV antibody), which demonstrated a ten-fold increase in FcRn binding affinity compared to the wild-type Fc (Dall'Acqua et al., 2006). Building upon this, additional substitutions (A530V, D671E, L673M) have been explored to further augment FcRn interaction (Zheng et al., 2020). Furthermore, an Fc-engineering approach, based on three amino acid substitutions (Q311R, M428E, N434W) improves pH-dependent human FcRn binding, which could not only prolong the plasma half-life but also potentiate complement-mediated killing or phagocytosis of cancer cells or bacteria (Foss et al., 2024).

Compared to monoclonal antibodies (mAbs)—which dominate the therapeutic biologics market in both number of approvals and commercial value—Fc fusion proteins offer a distinct and complementary set of pharmacological advantages. Their main advantage lies in extending the *in vivo* half-life of therapeutic components, which naturally have a relatively short half-life (such as receptor extracellular domains, peptides, or endogenous proteins) (Huang, 2009). In addition to enhanced pharmacokinetics, Fc fusions are able to offer unique molecular versatility. They can be engineered to act as “trap” molecules to neutralize disease-related ligands, such as aflibercept. It is a fusion protein constructed from the second domain of VEGFR-1 and the third domain of VEGFR-2 linked to the Fc fragment of human IgG1 (Chu, 2009). This structural design enables high-affinity binding and potent neutralization of circulating VEGF molecules. Furthermore, Moreover, Fc fusions can serve as scaffolds to present peptides or complex, multi-chain receptor architectures, as exemplified by romiplostim (Broudy and Lin, 2004). However, it is important to note that the half-life of Fc fusion proteins is highly variable and not universally superior to mAbs.

In 1998, the FDA approved the first Fc fusion therapeutic biomolecule, etanercept, for the treatment of rheumatoid arthritis (RA) and ankylosing spondylitis (AS). To date, the FDA has approved several drugs, including tumors, autoimmune diseases, hematologic disorders, inflammatory diseases, and transplant rejection (Table 1).

**TABLE 1:** Approved long-acting recombinant fusion proteins in recent years (exclude withdrawn drugs).

Name	Therapeutic protein	Long-term strategy	Application	Half-life	Approval year	References
Etanercept	TNFR2	Fc	Rheumatoid arthritis, Ankylosing spondylitis	102 h	1998 (FDA)	Epstein (1997), Zhou et al. (2011)
Abatacept	CTLA4	Fc	Rheumatoid arthritis	13.1 (8–25) d	2005 (FDA)	Ruderman and Pope (2005)
Rilonacept	IL1R	Fc	Cryopyrin-associated periodic syndromes	8.6 days	2008 (FDA)	Hoffman (2009)
Romiplostim	TPO-like peptide	Fc	Chronic immune thrombocytopaenic purpura	3.5 days (median)	2008 (FDA)	Cines et al. (2008)
Corifollitropin alfa	FSH	CTP	Female infertility	79 (59–82) h	2010 (EMA)	Croxtall and McKeage (2011)
Belatacept	CTLA4	Fc	Organ transplant rejection	9.8 days (10 mg/kg) 8.2 days (5 mg/kg)	2011 (FDA)	Vin et al. (2011)
Aflibercept	VEGFR	Fc	Neovascular age-related macular degeneration	7.13 days	2011 (FDA)	Stewart et al. (2012)
Ziv-aflibercept	VEGFR	Fc	Advanced colorectal cancer	6 (4–7) d	2012 (FDA)	Clarke and Hurwitz (2013)
Conbercept	VEGFR	Fc	Wet age-related macular degeneration	4.2 days (rabbits)	2013 (CFDA)	Wang et al. (2013), Li et al. (2012)
Efmoroctocog alfa	FVIII	Fc	Hemophilia A	19–20.9 h (>18 years) 16–17.5 h (12–18 years) 12.3–15.9 (<12 years)	2014 (FDA)	Frampton (2016)
Eftrenonacog alfa	FIX	Fc	Hemophilia B	77.6 h (>19 years) 66.49–82.22 (<18 years)	2014 (FDA)	Peters et al. (2010)
Dulaglutide	GLP-1 analogue	Fc	Type 2 diabetes	3.75 days (89.9 h)	2014 (FDA)	Sanford, 2014; Amblee (2016)
Asfotase alfa	Enzyme (PPi)	Fc	Hypophosphatasia	5 days	2015 (FDA)	Scott (2016)
Albutrepenonacog alfa	FIX	HSA	Hemophilia B	92 h	2016 (FDA)	Lyseng-Williamson (2017)
luspatercept	ActRIIB	Fc	Anaemia	11 days	2019 (FDA)	Markham (2020)
Telitacicept	TACI	Fc	B cell-mediated autoimmune diseases	11.8 days	2021 (NMPA)	Dhillon (2021)
Somatrogon	hGH	CTP	Growth hormone deficiency	28.3 h	2021 (TPD)	Lamb (2022)
Ozoralizumab	TNFα-binding domain	ABD	Rheumatoid arthritis	18.2 days	2022 (PMDA)	Tanaka, 2023; Keam (2023)
Efanesoctocog alfa	FVIII	XTEN144/288 +Fc	Hemophilia A	39.9 h (1–6 years) 42.4 h (6–12 years) 44.6 h (12–18 years) 48.2 h (>18 years)	2023 (FDA)	Seth Chhabra et al. (2020), Konkle et al. (2020)
Efbemalenograstim alfa	G-CSF	Fc	Chemotherapy-induced neutropenia	35.6 h	2023 (NMPA)	Blair (2023)
Nogapendekin alfa inbakicept	IL-15R	Fc	Non-muscle invasive bladder cancer	20 h	2024 (FDA)	Keam (2024)
Sotatercept	ActRIIA	Fc	Pulmonary arterial hypertension	24 days	2024 (FDA)	Kang (2024)
Efsubaglutide alfa	GLP-1	Fc	Type 2 diabetes	182–215 h	2025 (NMPA)	Lou et al. (2025)
Albipagrastim alfa	G-CSF	HSA	Chemotherapy-induced febrile neutropenia	34.8 h	2025 (NMPA)	Wang B. et al. (2025)

TNFR: p75-tumor necrosis factor receptor; CTLA4: the extracelluar domaine of cytotoxic T lymphocyte–associated protein 4; IL: interleukin; TPO: thrombopoietin; CTLA-4: cytotoxic T-lymphocyte-associated antigen 4; VEGFR: vascular endothelial growth factor receptors; TACI: transmembrane activator and calcium modulator and cyclophilin ligand interactor; FVIII: coagulation factor VIII; FIX: coagulation factor IX; GLP-1: glucagon-like peptide 1; ActR: activin receptor; G-CSF: Granulocyte colony-stimulating factor; FDA: U.S., food and drug administration; CFDA: china food and drug administration; NMPA: national medical products administration; PMDA: pharmaceuticals and medical devices agency; EMA: european medicines agency; TPD: Health Canada’s Therapeutic Products Directorate.

### 4.2 Human serum albumin, HSA

Human serum albumin (HSA), the most abundant plasma protein primarily synthesized by the liver, serves critical roles in maintaining osmotic pressure and transporting a wide variety of endogenous and exogenous ligands. Notably, HSA shares with the Fc domain of IgG a common recycling mechanism mediated by the neonatal Fc receptor (FcRn). The affinity of FcRn for HSA decreases approximately 200-fold upon transition from acidic to neutral pH, and the FcRn-albumin interaction is characterized by rapid association and dissociation kinetics. Through this pH-dependent salvage pathway, FcRn protects both albumin and IgG from lysosomal degradation, thereby significantly extending their plasma half-lives (Chaudhury et al., 2006; Chaudhury et al., 2003). The feasibility of harnessing this mechanism for therapeutic applications was first demonstrated by Yeh et al., in 1992, who engineered an HSA-CD4 fusion protein to block HIV entry into CD4^+^ cells. In rabbits, this fusion construct exhibited a half-life comparable to that of native HSA and approximately 140-fold longer than that of soluble CD4 alone (Yeh et al., 1992). This seminal work established genetic fusion to HSA as a viable strategy for extending the systemic persistence of therapeutic peptides and proteins.

Engineered HSA variants with enhanced FcRn affinity contribute to improved plasma half-life. A proline substitution at position 573 (K573P) in HSA boosts its binding affinity to the FcRn receptor by over 12-fold at acidic pH, significantly extending its serum half-life across species. This engineered HSA variant maintains its enhanced pharmacokinetic properties even when fused to therapeutic proteins, positioning it as a highly promising universal platform for extending the half-life of diverse biotherapeutics (Andersen et al., 2014).

The first approved HSA fusion protein on the market was albiglutide (Tanzeum), a GLP-1-HSA fusion developed for type 2 diabetes mellitus (Poole and Nowlan, 2014). Despite regulatory approval, it failed to achieve commercial success against competing products utilizing Fc fusion or fatty-acid acylation technologies with comparable efficacy, leading to its voluntary discontinuation in 2017. A subsequent innovative drug, albutrepenonacog alfa, was developed by fusing recombinant coagulation factor IX with human serum albumin (HSA). It effectively prevents bleeding and features an ultra-long half-life of up to 102 h, which is 3–5 times that of traditional recombinant FIX products. With an approved dosing regimen of once every 7 days, it significantly improves treatment adherence and quality of life for patients with hemophilia B (Lyseng-Williamson, 2017). More recently, albipagrastim alfa, a novel HSA-fused granulocyte colony-stimulating factor (G-CSF), has recently been approved in China. Its efficacy is non-inferior to that of PEGylated recombinant human G-CSF (PEG-rhG-CSF), with a comparable safety profile (Wang B. et al., 2025).

Compared to Fc-fusion therapeutics, the number of approved HSA-fusion drugs is considerably lower. This disparity stems from two major challenges associated with full-length HSA: its intricate, disulfide bond-rich structure (17 bonds) and competition from endogenous albumin for the FcRn recycling pathway. To circumvent these hurdles, a promising strategy involves genetically fusing the drug to a small, engineered albumin-binding domain (ABD), a topic that will be elaborated on in the next section.

### 4.3 Albumin-binding domain (ABD)

The Albumin-Binding Domain (ABD) is a compact, 46-amino-acid protein derived from surface proteins of certain streptococcal strains. It exhibits a remarkably high affinity for HSA (Jonsson et al., 2008), enabling ABD-fusion proteins to associate non-covalently with endogenous albumin *in vivo*. By hijacking the FcRn-mediated recycling pathway of albumin, this strategy confers prolonged plasma persistence upon therapeutic proteins without requiring direct fusion to full-length HSA (Andersen et al., 2011).

The versatility of the ABD platform has been demonstrated through various preclinical applications. A cyclic fusion protein (c-IFN-ABD) was developed via head-to-tail macrocyclization of an ABD fused IFN-α. This cyclic configuration preserved potent albumin-binding affinity while conferring enhanced stability; importantly, it achieved superior tumor penetration and retention, resulting in significantly improved antitumor efficacy compared to both linear counterparts and unmodified interferon (Guo et al., 2020). Similarly, another ABD-G-CSF fusion protein maintained near-native structural integrity and biological activity *in vitro*, while demonstrating substantially extended serum half-life (9.3 h vs. 1.7 h for unmodified G-CSF) and effective neutrophil recovery in a neutropenic rat model (Nikravesh et al., 2022).

The clinical translatability of ABD technology is exemplified by ozoralizumab (Nanozora), a novel TNF inhibitor approved in Japan in 2022. This 38 kDa trivalent nanobody potently neutralizes TNFα and uniquely incorporates a human serum albumin-binding domain, enabling convenient 4-week dosing intervals for rheumatoid arthritis treatment (Tanaka, 2023).

### 4.4 Transferrin, Tf

Transferrin is an iron-binding glycoprotein primarily synthesized in the liver, where it plays a central role in systemic iron transport and homeostasis. Transferrin undergoes clathrin-dependent, receptor-mediated endocytosis upon binding to the transferrin receptor (TfR), followed by pH-dependent iron release and subsequent recycling of the Tf-TfR complex back to the cell surface. This recycling mechanism confers upon transferrin a relatively long plasma half-life (Strohl, 2015; Chen et al., 2011).

The Tf recycling pathway has been harnessed for half-life extension through genetic fusion to therapeutic proteins. For example, GLP-1 was genetically fused to a non-glycosylated human Tf, creating a novel GLP-1-Tf fusion protein with peptidase resistance and a half-life approximately 2 days (vs. 1–2 min for native GLP-1) (Kim et al., 2010). Besides, the Proinsulin-Transferrin (ProINS-Tf) fusion protein functions as a liver-specific prodrug. Following administration, this construct undergoes slow conversion to active Insulin-Transferrin (INS-Tf) primarily within hepatocytes, mediated by TfR-mediated uptake. This unique mechanism results in a prolonged plasma half-life, sustained hypoglycemic effect, and selective action on hepatic glucose production while avoiding unintended insulin receptor activation in muscles, thereby reducing potential systemic side effects (Wang et al., 2014).

Due to the high molecular weight of Tf, a higher drug dose is required to achieve the same therapeutic molar concentration as non-conjugated proteins. Therefore, application of Tf fusion technology necessitates careful consideration of increased drug load to mitigate potential dose-related adverse effects.

### 4.5 Carboxyl terminal peptide, CTP

Thyroid-stimulating hormone (TSH) and the three gonadotropins—follicle-stimulating hormone (FSH), luteinizing hormone (LH), and chorionic gonadotropin (CG)—are heterodimeric glycoprotein hormones that share a common alpha subunit but possess distinct beta subunits conferring their unique biological activities (Matzuk et al., 1990). Among these, CG exhibits a significantly prolonged half-life attributed to a unique, highly sialylated 31-amino-acid C-terminal peptide (CTP) on its beta subunit. This peptide carries a strong negative charge and enhances sialylation, thereby increasing hydrodynamic radius and reducing renal clearance (Fares et al., 1992).

Corifollitropin alfa (FSH-CTP, Elonva®) was the first CTP-fusion protein to enter clinical trials and be approved (EMA, 2010) as a long-acting fertility treatment. It consistently demonstrates an approximately twofold longer half-life than recombinant FSH, regardless of subcutaneous or intravenous administration (Croxtall and McKeage, 2011). Besides Elonva, Somatrogon (NGENLA®), a once-weekly, long-acting human growth hormone utilizing CTP technology, has been approved (first in Canada, 2021) for pediatric growth hormone deficiency, offering non-inferior efficacy with a significantly reduced dosing frequency compared to daily injections (Lamb, 2022).

### 4.6 New technologies in recent years

A novel *in vivo* antibody-selective conjugation technology was designed to extend the half-life of peptide drugs. Kitahara et al. engineered an electrophilic peptide based on the Z33 domain (derived from Protein A), which enables site-selective covalent binding to circulating native IgG antibodies directly in living organisms (Kitahara et al., 2026). Attaching therapeutic peptides to endogenous IgGs significantly prolongs the drug’s pharmacokinetic profile. *In vivo* studies in wild-type and obese mouse models demonstrated that a single subcutaneous injection of the GLP-1 conjugates resulted in sustained body weight loss and improved blood glucose management for 10–15 days (Kitahara et al., 2026).

### 4.7 Comparative assessment of different fusion protein platforms

The immunogenic risks associated with different fusion protein strategies vary considerably. Fc, HSA, and CTP fusion proteins exhibit relatively manageable immunogenicity profiles, attributable to their human-derived structural origins or glycan-mediated epitope shielding. In contrast, the albumin-binding domain (ABD), originating from Streptococcal protein G, carries a higher baseline immunogenic risk due to its bacterial provenance, necessitating engineering optimization—such as truncation or de-immunization strategies—to mitigate potential immunogenicity. Transferrin (Tf), despite being an endogenous human protein with low intrinsic immunogenic risk, possesses an inherent immunomodulatory function—binding to the T-cell receptor alpha constant region and inhibiting T-cell activation—which may indirectly influence the overall immunogenicity profile of Tf-based fusion constructs.

In summary, compared to other protein fusion strategies for half-life extension, the Fc fusion platform offers distinct advantages in terms of clinical validation, manufacturing efficiency, and functional versatility. First of all, Fc fusion proteins have the longest history of clinical use, which provides a well-understood safety and efficacy profile that regulators and developers find reassuring. Moreover, Fc fusions leverage the same well-established manufacturing platforms as monoclonal antibodies, including high-yield CHO cell expression and protein A affinity chromatography purification. Compared with other platforms, this shared infrastructure reduces process development costs and timeline, decreasing manufacturing complexity. Besides, Fc fusion proteins exhibit well-conserved FcRn binding across different species, which have more established preclinical evaluation models than HSA fusion proteins (Andersen et al., 2013). As for stability and biophysical properties, the Fc domain provides stabilizing effects while alternative platforms may not exhibit equivalently and even introduce new challenges such as the strong negative charge of CTP fusions potentially impacting biological activity. Therefore, while alternative fusion technologies continue to advance and may offer specific advantages for particular applications, Fc fusion remains the platform of choice due to its proven clinical track record, manufacturing compatibility, mature preclinical evaluation models, and well-characterized safety profile.

## 5 Biodegradable synthetic polymers

PEGylation extends half-life primarily by increasing the hydrodynamic volume of therapeutic proteins to reduce renal filtration, and by sterically shielding the drug from proteolytic degradation and immune recognition. Despite its established role as a cornerstone technology for improving pharmacokinetic profiles, widespread clinical application has revealed several significant drawbacks. These include the increasing prevalence of pre-existing and treatment-induced anti-PEG antibodies, which can accelerate clearance and reduce efficacy; long-term toxicity concerns arising from tissue accumulation of non-biodegradable PEG polymers; and inherent complexities in manufacturing and analytical characterization associated with polydisperse conjugation products. To overcome the limitations of conventional polymer conjugation, researchers are increasingly exploring biodegradable synthetic polymers and unstructured polypeptides as next-generation alternatives. These approaches aim to retain the mechanistic advantages of PEGylation while addressing concerns related to immunogenicity, tissue accumulation, and manufacturing heterogeneity.

A glycine-rich homo-amino-acid polymer (HAP) consisting of 200 residues with the repetitive sequence (Gly_4_Ser)_n_ was genetically fused to the light chain of an anti-HER2 Fab fragment (4D5) (Schlapschy et al., 2007). The resulting fusion protein exhibited binding affinity for the HER2 antigen indistinguishable from that of the unmodified Fab fragment, indicating that HAP fusion did not interfere with antigen recognition. Biophysical characterization revealed an approximately 120% increase in hydrodynamic volume compared to the parental Fab, translating to a significantly extended plasma half-life of approximately 6 hours—three-fold longer than that of the unmodified fragment (Schlapschy et al., 2007). While the current pharmacokinetic profile remains inferior to that of mature PEGylation technology, the HAP platform offers distinct advantages, including one-step production via genetic engineering, simplified manufacturing processes, and inherently homogeneous products. Future optimization of HAP sequences through genetic engineering holds promises for achieving pharmacokinetic properties comparable to those of PEGylated therapeutics.

Gelatin, a mixture of high molecular weight peptides derived from hydrolyzed collagen, contains repeating Gly-Xaa-Yaa triplets (with Xaa and Yaa often being proline) (Thyagarajapuram et al., 2007). Its inherent hydrophilicity stems from abundant exposed hydrogen bonds and a high content of hydrophilic amino acids. While natural gelatin has diverse uses, recombinant gelatin offers superior tunability for specific applications through intentional sequence engineering. For instance, fusing an artificial gelatin-like protein (GLK) to granulocyte colony-stimulating factor (G-CSF) produced a chimeric GLK/G-CSF fusion protein, which exhibited a plasma half-life of ∼10 h—a nearly 6-fold extension over the unmodified G-CSF (∼1.76 h) (Huang et al., 2010).

Biodegradable synthetic polymers can be conjugated to therapeutic proteins through either recombinant or chemical methods to enhance biological half-life and/or solubility. While the specifics of chemical coupling processes fall outside the scope of this discussion, the rationale for biodegradable polymer adoption warrants consideration.

These polymers are specifically engineered to address the long-term accumulation and toxicity concerns associated with traditional non-degradable polymers such as PEG. They undergo controlled *in vivo* degradation into non-toxic small molecules, thereby mitigating tissue accumulation risks. While preserving the core function of half-life extension and potentially reducing immunogenic risk, several considerations remain. The safety profiles of degradation byproducts and residual immunogenicity require comprehensive validation, and manufacturing processes demand stringent control to ensure product homogeneity.

## 6 Unstructured polypeptides

Unstructured polypeptides, also known as intrinsically disordered polypeptides (IDPs) or random coil polymers, are synthetic or recombinant protein segments that lack a stable, folded three-dimensional structure under physiological conditions. Unlike traditional globular proteins, their conformational flexibility and highly hydrophilic nature make them valuable tools in biopharmaceutical engineering, particularly for optimizing the pharmacokinetic and physicochemical properties of therapeutic proteins and peptides. They address critical limitations of first-generation technologies, offering a biodegradable, low-immunogenicity, and precisely engineerable solution for half-life extension. Representative and widely adopted examples include XTEN, PAS, ELP, and PsTag.

### 6.1 XTEN polypeptide

XTEN represents a kind of unstructured hydrophilic, biodegradable protein polymers which can enhance the stability and solubility of peptide drugs. The first variant, XTEN864, comprising 864 amino acid residues, was developed in 2009 (Schellenberger et al., 2009). Its amino acid composition was strategically selected to avoid residues associated with structural or stability issues: hydrophobic residues (F, I, L, M, V, W, Y) were omitted to prevent aggregation; amide-containing side chains (N, Q) were avoided to ensure long-term storage stability; positively charged residues (H, K, R) were excluded to minimize nonspecific membrane binding; and cysteine (C) was eliminated to avoid disulfide-mediated heterogeneity. The final sequence consists of randomly arranged segments containing 8% alanine (A), 12% glutamate (E), 18% glycine (G), 17% proline (P), 28% serine (S), and 17% threonine (T). This composition confers high solubility, stability, and absence of secondary structure or aggregation propensity.

The physicochemical properties of XTEN directly contribute to its pharmacokinetic benefits. Its strong negative charge promotes electrostatic repulsion with the glomerular basement membrane, while its flexible, random-coil conformation provides an expanded hydrodynamic volume. These combined effects significantly reduce renal filtration, thereby extending plasma half-life. Furthermore, the modular design of XTEN allows precise tuning of pharmacokinetics by varying polymer length. Subsequent variants such as XTEN1008, XTEN576, XTEN432, XTEN288, and XTEN144 have since been developed and incorporated into fusion protein constructs for tailored therapeutic applications (Schellenberger et al., 2009; Zhou et al., 2024).

Efanesoctocog alfa (marketed as Altuviiio® by Sanofi-Sobi) is a novel recombinant factor VIII (FVIII) therapeutic. It consists of a B-domain-deleted single-chain FVIII molecule fused to the D’D3 domain of von Willebrand factor (VWF), with two XTEN linkers incorporated to optimize its structure: one XTEN288 connects the heavy and light chains of FVIII, and a second XTEN144 links the Fc domain to the VWF D’D3 domain (Seth Chhabra et al., 2020). This design exhibits a mean half-life approximately three-to four-fold longer than that of conventional recombinant factor VIII (Konkle et al., 2020). Supported by this significantly extended pharmacokinetic profile, it received regulatory approval in 2023 for the treatment of inherited hemophilia A in both adults and children in the United States and Japan.

VRS-317, another protein therapeutic employing XTEN technology, consists of a recombinantly fused human growth hormone (rhGH) flanked by two XTEN sequences: a longer XTEN segment at the N-terminus and a shorter XTEN segment at the C-terminus. This configuration is designed to simultaneously reduce glomerular filtration (via the N-terminal XTEN) and limit receptor-mediated clearance (via the C-terminal XTEN), thereby extending its plasma half-life in preclinical models (Cleland et al., 2012; Yuen et al., 2013). Currently, the drug has completed Phase III clinical trials for the treatment of growth hormone deficiency.

In type 2 diabetes therapeutics, a novel GLP-1 fusion construct (GLP-ABD-XTEN144) incorporating both an albumin-binding domain (ABD) and XTEN144 demonstrated a prolonged half-life of 12.9 h in mice, accompanied by significant glucose-lowering effects and reduced food intake (Zhou et al., 2024). Similarly, fusion of XTEN864 to the C-terminus of GLP2-2G yielded a conjugate with substantially prolonged activity, exhibiting a half-life of approximately 120 h in non-human primate studies and enabling markedly reduced dosing frequency (Alters et al., 2012).

### 6.2 PAS polypeptide

Building on the XTEN polypeptide framework, Schlapschy et al. refined the amino acid composition to better maintain a disordered, soluble structure. Threonine (Thr) was excluded because of its strong tendency to promote β-sheet formation, while glycine (Gly) was omitted due to its increasingly poor solubility in longer chains and its role in compacting the random-coil conformation desired for unstructured polypeptides (Schlapschy et al., 2013). As a result, the team selected proline (Pro), alanine (Ala), and serine (Ser) as the preferred building blocks. Although homopolymers of each amino acid individually favor distinct secondary structures, a strategically designed sequence incorporating all three can counterbalance their respective structural propensities (Schlapschy et al., 2013). Further analysis revealed that Pro and Ala were the primary structural drivers of the polypeptide’s favorable biophysical properties. Consequently, the resulting sequence was designated as proline/alanine-rich sequences (PAS). This deliberate arrangement disrupts regular folding, thereby promoting a stable, irregular, and predominantly disordered conformation. PAS represents a sophisticated, genetically encoded solution for half-life extension, and is able to fuse to therapeutic proteins to significantly improve their pharmacokinetic properties.

Indeed, several PASylated biopharmaceuticals have been generated up to now. Vascular Endothelial Growth Factor A (VEGFA) overexpression is closely associated with pathological angiogenesis in diseases such as cancer, age-related macular degeneration (AMD), diabetic retinopathy, and other neovascular disorders. A novel anti-VEGFA nanobody fused with a PAS polypeptide demonstrated significantly improved physicochemical stability and pharmacokinetic performance, achieving an approximately 14-fold extension in plasma half-life in murine studies (Khodabakhsh et al., 2018; Khodabakhsh et al., 2020). Leptin plays a central role in appetite regulation. To overcome its short *in vivo* circulation time, leptin was fused with a PAS polypeptide to increase its hydrodynamic volume and delay renal clearance. Compared with the unmodified leptin, this fusion extended the plasma half-life in mice from 26 min to 19.6 h (Morath et al., 2015). Interferon-β1b (IFN-β1b) is a recombinant cytokine widely used as a disease-modifying therapy for relapsing forms of multiple sclerosis, though its efficacy is limited by the poor solubility, the weak stability and the short half-life in systemic circulation. PASylated-IFN-β1b exhibited a 2-fold increase *in vitro* biological activity, as well as significantly enhanced stability and solubility in solution compared to the unmodified protein (Zvonova et al., 2017). In addition, the PASylated-IFNα, aiming to clearing hepatitis B virus, effectively inhibits virus replication *in vitro* and reveals better *in vivo* antiviral effects than the unmodified ones (Xia et al., 2019). Recombinant erythropoietin (EPO) is responsible for the anemia related to the chronic kidney disease or cancer therapy, with a 4- to 13-h half-life following intravenous administration. While the PASylated EPO shows reduced *in vitro* potency, its *in vivo* activity is considerably increased on account of the 15.6-fold plasma half-life extension in comparison to the epoetin α (Hedayati et al., 2017). Furthermore, for peptide drugs with poor water solubility, a combined strategy of fatty acid modification and PAS modification can be employed to simultaneously enhance hydrophilicity and extend plasma half-life (Wang C. et al., 2025).

### 6.3 PsTag

Similar to XTEN and PAS polypeptides, many amino acids prone to aggregation or complex structure formation were first excluded, followed by the construction of a library consisting of 10-amino-acid fragments. The library was designed under two guiding principles: first, consecutive identical amino acids were prohibited; second, each fragment followed a fixed compositional ratio: two proline (P), three serine (S), two threonine (T), one alanine (A), and two glycine (G) residues (PsTag) (Yin et al., 2016). After coupling candidate fragments with GFP, two candidate peptides yielding the most distinct fluorescence were screened out, subsequently leading to the generation of PsTag200, PsTag216, PsTag400, and PsTag600 variants through self-connection (Yin et al., 2016; Yin et al., 2019).

The PsTag-FGF21 fusion protein, generated by linking PsTag600 to FGF21, demonstrated sustained pharmacological activity in mice and served as an effective therapeutic candidate for metabolic dysfunction-associated steatohepatitis (MASH) (Yin et al., 2018; Ji et al., 2024b). Another PsTag-modified protein is a complex delivery system made from PsTag polypeptide, polyglutamic acid chain, a matrix metalloproteinase 2 (MMP2)-degradable domain and a cationic cell-penetrating peptide, which exhibits enhanced tumor-targeted delivery, reduced off-target toxicity, and improved pharmacokinetic properties, while maintaining potent anticancer activity (Yin et al., 2019).

### 6.4 Elastin-like polypeptides (ELPs)

Elastin is a key extracellular matrix protein that provides elasticity and resilience to tissues such as skin, blood vessels, and lungs, enabling them to stretch and recoil. Elastin-like polypeptides (ELPs), derived from the natural elastin precursor, are a class of synthetic polypeptides imitating the repeat sequences of (VPGXG)n, in which X can be replaced by any amino acid residue except proline (Urry, 1999). The most distinctive property of ELPs is their inverse temperature transition: above a transition temperature (Tt), they undergo reversible phase separation into an insoluble and condensed coacervate phase (Smit et al., 2015). Notably, Tt can be precisely programmed by tuning the identity of the “X” residues in the pentapeptide repeat, the overall molecular weight of the ELP, or the physicochemical conditions of the solution (Urry, 1992; Fletcher et al., 2019). In addition, ELPs have been engineered for protein conjugation owing to their excellent biocompatibility, low immunogenicity, and susceptibility to degradation by endogenous collagenases (Gong et al., 2022). Leveraging these properties, ELPs of tailored lengths are fused to therapeutic proteins or peptides; their intrinsic ability to form micelles *in vivo* is then harnessed to achieve sustained drug release.

After being fused with ELP, the GLP-1-based therapeutic for type 2 diabetes demonstrated significantly prolonged glucose-regulating efficacy in a mouse model. A single administration achieved stable blood glucose reduction lasting up to 5 days, representing an approximately 120-fold extension in duration compared to the native GLP-1 peptide (Amiram et al., 2013). FGF21 has emerged as a promising therapeutic target for type 2 diabetes due to its ability to enhance systemic insulin sensitivity, making it an ideal candidate for combination therapy with GLP-1 receptor agonists. To overcome their inherently short plasma half-lives, an ELP was incorporated into the design of a dual-agonist molecule, which serves dual functions: it acts as a flexible linker between the GLP-1 and FGF21 moieties, while also providing sustained release properties, thereby extending dosing intervals to once weekly (Gilroy et al., 2020).

IFNα, a potent inhibitor of tumor cell proliferation, was genetically fused at its C-terminus to an ELP with the sequence (VPGXG)_90_ to prolong its therapeutic exposure. In this ELP design, the “X” position is occupied by alanine (A), glycine (G), and valine (V) in a molar ratio of 2:3:5. The resulting IFNα-ELP fusion achieved a 27.7-fold extension in plasma half-life, increasing from 0.3 h to 8.6 h (Hu et al., 2015). To further extend the circulation duration of IFNα-ELP and enable its long-term release, Wang et al. designed IFNα-ELP(V) by fusing IFNα with an ELP sequence of (VPGVG)_90_. Following a single subcutaneous injection in a mouse model, the fusion protein formed an *in situ* depot that achieved zero-order sustained release over 1 month. This delivery system demonstrated significantly enhanced tumor accumulation and eradication, along with substantially improved tolerability and biosafety compared to unmodified IFNα (Wang et al., 2018). Furthermore, the IFNα fusion protein incorporating an ELP diblock copolymer demonstrated a circulation half-life of 54.7 h (Wang et al., 2020).

The unstructured peptide platform is reshaping the technological landscape for the long-term delivery of therapeutic biomolecules through its biodegradable, low immunogenicity, and highly customizable advantages. It is not only a powerful alternative to PEGylation, but also becomes an important part in the development of next-generation long-acting biologics due to its ability to precisely control pharmacokinetics through rational design. However, the immunogenicity induced by non-native repetitive sequences within unstructured polypeptides warrants continued attention upon clinical translation.

## 7 Discussion

**FIGURE 3:**
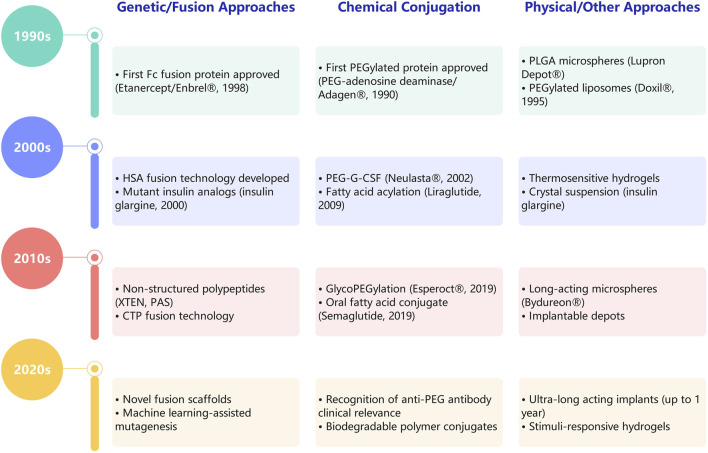
Key milestones in half-life extension strategies for protein therapeutics. Major developments are categorized into three strategy families: Genetic/fusion approaches (left), Chemical conjugation (middle), and physical/other approaches (right). Each decade (1990s–2020s) highlights representative approved drugs, technological breakthroughs, and emerging trends.

Over recent decades, strategies for extending the half-life of recombinant protein therapeutics have evolved into three primary categories: chemical conjugation (e.g., PEGylation), physical approaches (e.g., nanoparticle encapsulation) and genetic fusion to endogenous long-circulating carriers (e.g., Fc fragment, HSA, transferrin) or unstructured polypeptides (e.g., XTEN, PAS) (Figure 3). These innovations have been instrumental in shifting treatment paradigms from daily to weekly or even monthly administrations, significantly enhancing patient compliance and clinical outcomes. Table 2 presents a systematic comparison of genetic/fusion approaches, chemical conjugation, and physical delivery systems across multiple dimensions. In this section, we critically analyze the relative merits and limitations of these strategies and discuss their implications for therapeutic protein development.

**TABLE 2:** Comparison of half-life extension strategies in protein therapeutics.

Strategy	Genetic/Fusion approaches (e.g., amino acid mutation, Fc fusion, HSA fusion)	Chemical conjugation (e.g., PEGylation)	Physical/Other approaches (e.g., drug delivery system)
Representative Techniques	1. Protein fusion 2. Non-structured polypeptides 3. Site-directed mutagenesis	1. Linear/branched PEGylation 2. GlycoPEGylation (site-specific) 3. Fatty acid conjugation 4. Polysialic acid (PSA) conjugation	1. Microspheres (PLGA, PLA) 2. Liposomes 3. Hydrogels 4. Implantable depots 5. Crystal suspensions
Mechanism	1. FcRn-mediated recycling 2. Increased hydrodynamic size 3. Protection from degradation 4. Altered pI for precipitation	1. Steric hindrance 2. Increased hydrodynamic size 3. Reversible albumin binding	1. Sustained release from depot 2. Protection from degradation
Feasibility	1. Requires molecular cloning 2. Single microbial or cell culture process 3. Standard downstream purification 4. Low product heterogeneity	1. Requires purification and conjugation chemistry 2. Reaction conditions must be optimized	1. Often requires complex formulation development 2. Formulation stability must be validated
Scalability	Scalable in CHO or *E. coli* systems	Scalable but requires additional steps (conjugation and purification)	Scalability varies; often more complex than standard protein production
*In Vitro* Performance	Typically retains full intrinsic activity if fusion or mutation site does not sterically hinder the active site	Risk of reduced potency due to random conjugation blocking active site; site-specific PEGylation can mitigate this	Protein remains unmodified and fully preserved
*In Vivo* Half-life Extension	1. 2–20 fold 2. Protein fusion: ∼1–2 weeks in humans 3. Non-structured polypeptide: tunable	1. 2–10 fold 2. PEG: size-dependent 3. Fatty acids: albumin-mediated (days)	1. Weeks to months 2. Microspheres: 1–6 months 3. Implants: up to 1 year
Immunogenicity	1. Human sequences: low risk 2. Junction or non-structured polypeptide: may produce new antigenicity	1. PEG might elicit anti-PEG antibodies 2. Fatty acids: low risk	Highly dependent on material; some polymers may provoke inflammatory responses
Advantages	1. Genetically encoded single production process 2. Exploits natural recycling (FcRn) 3. Homogeneous product	1. Broadly applicable to diverse drugs 2. Tunable PEG size for desired half-life 3. Fatty acids enable oral delivery potential	1. Ultra-long dosing intervals possible 2. Preserves native protein structure 3. Can deliver high drug loads
Limitations	1. Fusion partner may affect folding or activity 2. Limited to proteins that can be fused 3. Immunogenicity at fusion junctions	1. Product heterogeneity 2. Anti-PEG antibodies emerging concern 3. Conjugation may inactivate protein 4. Additional steps increase cost	1. Complex manufacturing 2. Burst release risk 3. Potential toxicity from carrier materials

### 7.1 Genetic or fusion approaches: balancing efficacy with molecular integrity

As the primary focus of this review, genetic or fusion strategies offer the unique advantage of producing a homogeneous, well-defined molecular entity through a single manufacturing process. A critical consideration emerging from our analysis is the trade-off between half-life extension and preservation of molecular integrity. While genetic approaches generally preserve *in vitro* potency when fusion sites are carefully selected, the addition of large fusion partners may occasionally sterically hinder active domains (Wang et al., 2016) or impair tissue penetration (Brandl et al., 2019) due to increased molecular size. Furthermore, although human-derived sequences such as Fc and HSA exhibit low intrinsic immunogenicity, the fusion junctions themselves may create neo-epitopes capable of eliciting immune responses, which should be concerned carefully during preclinical development (Schmidt et al., 2024).

The clinical success of numerous Fc fusion proteins (e.g., etanercept, abatacept) and the emergence of next-generation platforms such as XTEN and PAS polypeptides underscore the maturity and versatility of genetic approaches. However, their applicability remains constrained to proteins that can be successfully expressed as fusions in microbial or mammalian systems, excluding certain complex or aggregation-prone candidates.

### 7.2 Chemical conjugation: half-life extension at the cost of heterogeneity

Chemical Modification (e.g., PEGylation, GlycoPEGylation, Fatty Acid Conjugation) is highly versatile and can be applied to a wide range of proteins without requiring genetic engineering of the protein itself. Techniques like PEGylation are well-established, with several approved drugs, offering a proven path to regulatory approval. The modifications primarily increase the hydrodynamic radius of the protein, effectively reducing renal clearance.

A key insight from our comparative analysis is the critical importance of site-specificity in chemical conjugation. Traditional random PEGylation, while effective, often yields heterogeneous product mixtures (Fee, 2007; Fee and Van Alstine, 2004) and may conjugate near active sites, resulting in substantial loss of *in vitro* potency (Kubetzko et al., 2005). In addition, the emerging recognition of anti-PEG antibodies (Armstrong et al., 2007; Ganson et al., 2006) has caused other concern. The extra manufacturing steps required for conjugation and purification of modified protein also contribute to increased production costs and complexity compared to genetic approaches (Fee, 2007; Fee and Van Alstine, 2004).

### 7.3 Physical delivery systems: achieving ultra-long duration at the expense of complexity

Physical delivery systems—microspheres (Su et al., 2021), liposomes (Bardania et al., 2017), hydrogels (Griswold et al., 2022), implants (Agarwal and Rupenthal, 2013), and crystal suspensions (Home, 1999)—occupy a distinct niche in the half-life extension landscape, achieving the longest dosing intervals (weeks to months) while leaving the protein molecule itself completely unmodified. This preservation of native structure ensures full retention of *in vitro* potency and avoids the immunogenicity risks associated with molecular modification.

However, this exceptional duration comes at the cost of substantial manufacturing complexity. Unlike genetic and chemical approaches, which modify the drug molecule and can leverage standard protein production infrastructure, physical systems require specialized formulation development, aseptic processing of particles, and rigorous validation of release kinetics and stability. The material-dependent immunogenicity of physical systems also warrants consideration. While PLGA microspheres have an established safety profile, novel materials may provoke inflammatory responses, and liposomal formulations can activate complement, leading to infusion reactions. These considerations underscore the importance of careful material selection and thorough preclinical evaluation.

### 7.4 Several implications and future directions

The comparative analysis presented in Table 2 and discussed above carries several implications for therapeutic protein development. First, the choice among strategy categories should be guided by the specific characteristics of the drug candidate and the therapeutic context. Genetic/fusion approaches offer an attractive balance of significant half-life extension, product homogeneity, and manufacturing simplicity for proteins amenable to fusion. Chemical conjugation provides a versatile alternative for candidates where fusion is not feasible. Physical delivery systems, while most complex, are unparalleled for indications requiring ultra-long dosing intervals.

Besides, the boundaries between these categories are increasingly blurring, with hybrid strategies emerging as a promising direction. Examples include the encapsulation of genetically engineered long-acting proteins within microspheres, or the combination of amino acid mutation with fatty acid modification. Such approaches may offer synergistic benefits, achieving both molecular-level stabilization and better half-life extension.

In brief, the choice of a half-life extension strategy is not one-size-fits-all but requires a balance between pharmacokinetic goals, bioactivity, manufacturing complexity, and long-term safety data. A nuanced understanding of the relative strengths and limitations of genetic, chemical, and physical approaches will be essential for rational selection and combination of these strategies to achieve desired clinical outcomes.
